# Untargeted Lipidomics in Fabry Disease of Urine Samples by Low-Resolution Flow Injection Mass Spectrometry (ESI(±)-LTQ MS)

**DOI:** 10.1021/acsomega.5c00894

**Published:** 2025-06-27

**Authors:** Rafael Arruda Foletto, Augusto Santos Borges, LarissaCampos Motta, Marcos Valério Vieira Lyrio, Carolina Teles Baretto, Luciene Cristina Gastalho Campos, Paulo Roberto Filgueiras, Valério Garrone Barauna, Wanderson Romão

**Affiliations:** † Laboratory of Molecular Physiology and Artificial Intelligence, Department of Physiological Science, 28126 Federal University of Espírito Santo (UFES), Vitória, Espírito Santo 29075-910, Brazil; ‡ Federal Institute of Espírito Santo (IFES), Vila Velha, Espírito Santo 29106-010, Brazil; § Laboratory of Petroleomics and Forensic Chemistry, Chemistry Department, Federal University of Espírito Santo (UFES), Vitória, Espírito Santo 29075-910, Brazil; ∥ Postgraduate Program in Health Sciences, State University of Santa Cruz, Ilhéus, Bahia 45662-900, Brazil

## Abstract

Background: Fabry disease (FD) is a lysosomal storage disease caused by genetic mutations related to the coding of the enzyme α-galactosidase A, which is responsible for the metabolism of glycosphingolipids such as globotriaosylceramide and globotriaosylsphingosine. The accumulation of these and other metabolites can occur in various types of cells and impair the functioning of multiple organs and systems, such as the heart, brain, and kidneys. However, with early diagnosis and appropriate therapeutic intervention, the clinical outcome can be significantly improved. This study aimed to analyze the performance of new diagnostic methods for FD using the broad field of lipidomics combined with multivariate analyses, proposing the use of urine as a specimen. Materials and Methods: urine samples were collected from patients with both confirmed (Case) and negative (Control) diagnoses of FD, which were later processed for specific lipid extraction. After extraction, 81 samples (44 cases and 37 controls) were subjected to mass spectrometry analysis, with direct infusion and electrospray ionization in both positive and negative modes (ESI(±)). After spectral acquisition, the data were processed and analyzed using multivariate analysis methods such as Principal Component Analysis (PCA) and Partial Least Squares Discriminant Analysis (PLS-DA). Results: the combination of both ionization modes for PLS-DA was able to differentiate between the Case and Control groups with 92% accuracy. Conclusion: this paper suggests that the proposed method of application of lipidomics combined with multivariate analyses as a tool for early diagnosis of FD is promising, enabling and contributing to the improvement of healthcare for these patients.

## Graphical abstract

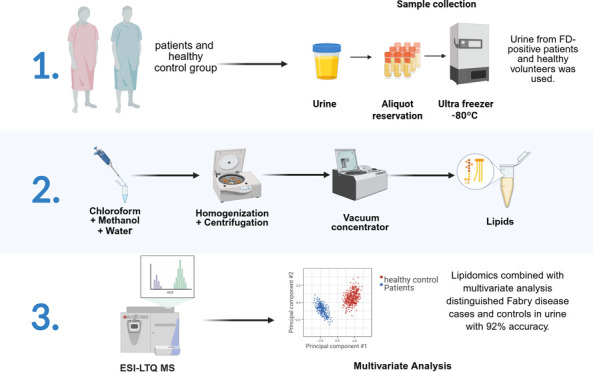

## 1 Introduction

Fabry disease (FD) is an X-linked lysosomal storage disease caused by a mutation in the gene encoding the enzyme α-galactosidase A (α-GAL A).^1^ A reduction or absence of α-GAL A activity results in the progressive accumulation of the substrates globotriaosylceramide (Gb3), globotriaosylsphingosine (lyso-Gb3), and related glycosphingolipids in various cell types, affecting multiple systems and afflicting both males and females.^2^ The incidence of FD varies between 1:40,000 and 1:117,000 births in the general population, although some screening studies point to higher incidence rates (1:1500–1:7000).^3^

The progressive accumulation of glycosphingolipid derivatives in lysosomes can occur in various tissues, such as endothelial, vascular, cardiac, renal, and nervous, leading to cell damage and organ failure, significantly reducing life expectancy. The classic phenotype of FD manifests as cornea verticillata, neuropathic pain, gastrointestinal dysfunction, and angiokeratoma. Serious complications typically occur in adulthood and may include progressive renal failure, cardiac complications (arrhythmia and hypertrophic cardiomyopathy), and central and peripheral nervous system involvement.^4^

In general, FD presents itself more severely in hemizygous males than in heterozygous females. However, due to random inactivation of the X chromosome, some women may be affected by FD as severely as men. Urinary Gb3 is used as a diagnostic biomarker of FD and to monitor response to treatment since urinary Gb3 levels are higher in FD patients than in healthy controls. However, normal urinary Gb3 levels cannot exclude the diagnosis of FD for heterozygous females or males with the late onset form of the disease. The diagnosis of FD should be confirmed by testing for α-Gal A activity and/or by detecting a disease-causing variant of the galatosidase α (GLA) gene. Pathogenic variants may present with low or absent residual α-Gal A activity in males, whereas heterozygous females have greater variability due to random inactivation of the X chromosome.^5^

The classic form of FD presents in early childhood with mild symptoms, whereas cardiovascular disease and renal failure occur in middle age, leading to a worse prognosis over the years.^6^ Regarding treatment, enzyme replacement therapy (ERT) with intravenous exogenous human galactosidase A significantly improved the care of FD by increasing serum levels of the enzyme in the body. At the same time, oral pharmacological chaperones, such as Migalastat, promote the correct folding of amenable mutated glycosides, regenerating levels of residual activity. Although they are in their early stages, substrate reduction therapy and gene editing therapies using adeno-associated viral vectors can also be highlighted since these innovative therapeutic approaches are currently being investigated and tested.^4^

Recent systemic analyses have shown that Gb3 is not the best biomarker for diagnosing the disease or assessing a patient’s response to treatment. When measured in random total urine samples, Gb3 levels may appear normal in some patients and in heterozygous women. Therefore, an alternative approach is needed for a timely diagnosis of FD, particularly in female patients.^6^ For better discrimination, lyso-Gb3, the deacylated form of Gb3, is suggested, although false negatives in some patients and in very late cases have been described.^4^

The metabolite lyso-Gb3 was discovered in 2008 as a more accurate biomarker of disease manifestation and progression. Since then, lyso-Gb3 has become a preferred biomarker for FD. Based on extensive research, it is now known that healthy individuals contain less than 1 nM lyso-Gb3 in plasma, but patients with classic and late onset phenotypes have 100 and 10 times higher concentrations, respectively. It is important to highlight that the quantification of lyso-Gb3 is best performed using more modern methods such as ultraperformance liquid chromatography coupled to tandem mass spectrometry (UPLC-MS/MS) with the use of an internal standard.^7^

An appropriate care of FD requires an early diagnosis. However, the lack of robust markers and a molecular understanding of the pathophysiology of FD hinders or delays effective diagnosis, patient stratification, and personalized care. Therefore, a better understanding of the biological plasticity of FD may reinforce screening and diagnostic tools. To this end, the omics sciences have revealed new perspectives for exploring biological systems through data-rich strategies with unprecedented breadth, depth, and reach in different areas, including lysosomal storage diseases.^4^

Moreover, the increased specificity of GLA gene testing is accompanied by high cost, uncertain negative predictive value when encountering genetic variants of uncertain significance (VUS), and the comparatively low cost of biomarker-based screening. Other than that, there is a lack of prospective values studies of one screening strategy over the other. An investigation of the cost efficacy of α-Gal A enzyme screening has revealed that, assuming a conservative cost estimate of 100 US dollars for α-Gal A enzyme analysis and the need for confirmatory GLA testing (estimated at 400 US dollars), the total estimated cost to screen and diagnose one new case of FD is approximately 24,000 US dollars in a cohort of 238 clinically suspected hypertrophic cardiomyopathy patients.^8^

### 1.1 Metabolomics and Lipidomics

Metabolomics is an investigative method with high-throughput technologies with the aim of analyzing an entire biological system at the moment that it is observed in an unbiased and hypothesis-free way. Untargeted metabolomics is devoted to the analysis of a large set of compounds with a more exploratory role and provides opportunities for new discoveries by linking cell signaling pathways with biological mechanisms to better understand cell biology and physiology. Targeted metabolomics, on the other hand, is typically used for the investigation of specific pathways that are altered at different stages of a disease or treatment. This approach is commonly guided by a specific biological question where a limited number of well-defined compounds are considered, is better suited for quantitative analysis, and requires the use of specific and quantitative analyses to provide meaningful and reliable answers.^4,9^

Metabolomics can be defined as the systemic characterization of metabolites and their fluctuations related to genetic and environmental factors. Technologies capable of comprehensively defining the biochemical profile of a given biological sample include mass spectrometry (MS), nuclear magnetic resonance (NMR), and Fourier-transform near-infrared spectroscopy (FT-NIR). MS is among the most widely used for both quantitative and qualitative analysis due to its high resolution, sensitivity, and specificity.^10–12^

Lipidomics is the branch of metabolomics consisting in the comprehensive study of lipid species and their related networks and pathways of a biological system.^13^ This approach enables the study of cellular metabolism by quantifying the differences between lipids that indicate metabolic variations. Specifically, it can aid in the early diagnosis of rare diseases or contribute to a better understanding of different manifestations of metabolic diseases.^14^ Due to the wide range of physiological functions of lipids, lipidomic characterization of affected individuals can not only identify new biomarkers but also improve the understanding of the pathophysiological mechanism and facilitate the monitoring of the disease evolution in the long term.^15^

Lipidomics has already proven useful in the clinical investigation and clinical diagnosis of rare diseases, while liquid chromatography–mass spectrometry (LC–MS)-based analyses are rapidly becoming the main technique in the discovery of new biomarkers in FD. However, although MS is routinely used in clinical laboratories, adding new assays to diagnostic tests requires additional validation that includes complementary sensitivity and specificity tests, as well as comparison to already established methods for diagnosing patients.^14^

Phenotypic diagnosis of FD requires combinations of clinical, biochemical, and molecular criteria, as well as biomarkers with the best available sensitivity and specificity, to classify individual patients in regards to disease severity, prognosis, and treatment management.^16^ In view of the urgent need for rapid and accurate diagnostic methods, the combination of biofluid analysis with MS technology presents itself as a promising strategy for the efficient screening of patients with FD, highlighting the agility in diagnosis, which is essential for the adoption of measures that prevent the devastating progression of the disease. In addition, the use of urine with modern analysis techniques such as LC–MS allows the analysis of a wide range of biomarkers, such as proteins, lipids, and their metabolites, which reflect biochemical changes induced by FD.^17^

Specifically for FD, metabolomic analysis is a valuable tool for understanding pathological processes at the molecular level and for identifying molecules capable of recognizing and discriminating many pathogenic phenotypes. The discovery of robust biomarkers of FD can help achieve an early diagnosis and a better stratification of patients for efficient therapeutic care and monitoring.^4^ Therefore, given the complexity of phenotypes and metabolic alterations in FD, the need for more biomarkers to complement serum lyso-Gb3 is highlighted, particularly for groups with cardiac variants and patients with genetic VUS.^18^

What is generally observed in certain analytical techniques is the generation of collinear data due to the formation of numerous dependent variables (repeated information), which makes it all the more difficult to build classification models and better visualize the data. Given the structural diversity of lipids, untargeted lipidomics approaches produce large and highly complex data sets that need to be further processed to obtain meaningful information. Due to the large amount of data generated, the integration of MS with statistical tools such as multivariate analysis becomes essential to reduce data complexity and increase metabolite coverage and throughput.^9,13^ This strategy represents a significant advance in the development of more effective diagnostic strategies, providing rapid and personalized interventions for better clinical management of FD, though even untargeted assays based on high-resolution MS may be difficult to establish in clinical practice.^19^ Among the existing multivariate analysis techniques, Principal Component Analysis (PCA) and Partial Least-Squares Discriminant Analysis (PLS-DA) stand out.

### 1.2 Multivariate Analysis

PCA is an unsupervised exploratory method with the objective of reducing the dimensionality of the original data set, preserving as much information as possible. This reduction is achieved through the establishment of new variables orthogonal to each other called principal components (PCs). Arranged in descending order of importance, the PCs are linear combinations of the original variables. The graphs obtained represent the samples in a Cartesian system, where the axes are the PCs. The evaluation of the PCs can help in the establishment of a particular chemical signature for each group of samples segregated after PCA.^20^ PCA is primarily used to obtain an overview of the degree of separation between the groups and, additionally, to observe the grouping between the different classifications in order to detect outliers in a data set.^17^

PLS-DA is a supervised method that optimizes the separation between different groups of samples by maximizing the covariance between independent variables and by creating latent variables that consist of linear combinations of the original variance. As it is a supervised method, PLS-DA takes into account the groups to which the samples belong, though overfitting may occur if the data set is small and validation may be necessary in order to confirm the strength of the test. The advantage of this approach is its ability to handle the highly collinear and noisy data characteristic of the LC–MS and NMR results. PLS-DA allows complex data sets to be visualized through easily interpretable scatter plots that can show the separation between groups.^17^

In view of the progressive and degenerative nature of FD, considering that the characteristic accumulation of lipids begins long before the onset of symptoms and with the need to start treatment as soon as possible, analytical methods capable of detecting specific biomarkers in low concentrations, in a timely fashion, in different biological matrices, and that allow distinguishing between different pathological variations are increasingly necessary. The analysis of lipids, especially glycosphingolipids, can also be applied as a measure to evaluate the therapeutic response since the reduction in the levels of some of these metabolites, in both urine and plasma, is indicative of the efficacy of the treatment. Therefore, this type of evaluation has the potential to provide pertinent information regarding the severity, progression, and response to treatment of the disease in question, in addition to allowing for a better stratification of patients. The objective of the present study was to perform the lipid profile of urine samples from patients with Fabry disease by means of low-resolution mass spectrometry with an LTQ (linear ion trap) analyzer combined with direct infusion by electrospray in positive and negative ionization modes (ESI(±)) with subsequent multivariate analysis of the acquired data.

## 2 Materials and Methods

### 2.1 Reagents

All reagents were obtained from Sigma-Aldrich Chemicals, Darmstadt, Germany, unless otherwise specified. The reagents used in the tests are as follows: methanol; chloroform; acetonitrile; formic acid; ammonium hydroxide (NH_4_OH); monobasic and dibasic sodium phosphate; and ultrapure water (18 MΩ, Direct-Q Merck, Darmstadt, Germany). The solvents were used as described in the procedures.

### 2.2 Study Population

The samples were provided by the Department of Physiological Sciences of the Federal University of Esprito Santo (UFES) and were collected as part of a previous study entitled “Análise de fluidos corporais de pacientes com doença de Fabry através da espectroscopiavibracional (ATR–FTIR)”, authored by the master researcher Carolina Teles Barretto and in cooperation with the State University of Santa Cruz (UESC). The study was previously approved by the Research Ethics Committee of the institution under protocol number CAAE 36894020.4.0000.5526 and opinion number 4.992.927. Prior to sample collection, all patients who participated in the study signed the Informed Consent Form (ICF). The samples collected from each participant included biofluids such as blood, urine, and saliva.^21^

The specimen chosen for this study was urine collected from patients with a positive diagnosis of FD (Case group) and from healthy volunteers with a negative diagnosis of FD (Control group). Between January 2021 and January 2022, 52 patients diagnosed with FD hailing from the Kidney Care Clinic, Ilhéus, BA, the Cassiano Antonio Moraes University Hospital, Vitória, ES, and other referring doctor’s offices were evaluated. The inclusion criteria were that the participants must have had a mutation of the GLA gene confirmed by genetic testing. The mutations were classified as VUS, classic, or late phenotype based on the Fabry-Gen-Phen (http://fabrygenphen.com/), OMIM (http://www.ncbi.nlm.nih.gov/omim/) and/or the H. Sakuraba (www.fabry-database.org) mutation database. Clinical data, imaging tests, and laboratory results were obtained from the medical histories of the patients. The Control group also consisted of 52 participants organized by sex and age.^21^

### 2.3 Sample Preparation

The lipid extraction method employed was an adaptation of the one proposed by Bligh and Dyer for simple and rapid extraction and purification of lipids from biological materials.^22^ For preparation, 190 μL of urine was collected from each sample, and 500 μL of chloroform, 500 μL of methanol, and 260 μL of 20 mM phosphate-saline buffer (pH = 8) were added to each aliquot.^23^ The samples were then vortex-homogenized for 1 min and then centrifuged for 10 min at 15,000*g* for separation. The organic phase (bottom) was collected, and another 500 μL of chloroform was added to ensure complete extraction, followed by homogenization for 1 min in vortex and centrifugation for 10 min at 10,000 G. The organic phase was collected and vacuum-evaporated in a benchtop concentrator model Concentrator plus (Eppendorf, Hamburg, Germany). Finally, the samples were resuspended in 200 μL of methanol, centrifuged for 10 min at 15,000*g*, and transferred to previously identified vials for further analysis.^22^ All samples were prepared in the Multiuser Center for Technological Development and Innovation of Vila Velha (CMVV) IFES laboratory and analyzed in the Multiuser Laboratory of Petroleum and Forensics/LabPetro on the Goiabeiras UFES campus.

### 2.4 ESI­(±)-LTQ MS Analyses

The LTQ MS analyses were performed in an LTQ XL Linear Ion Trap mass spectrometer (Thermo Fisher Scientific, Massachusetts, United States of America). For analysis, 5 μL of each sample was injected and eluted in methanol and introduced into the equipment by flux injection and electrospray ionization in positive and negative modes (ESI(±)). In the positive ionization mode, the methanol used was acidified with 0.1% formic acid, and for the negative mode, the methanol used was alkalized with 0.4% NH_4_OH.^15^ Solvent blank analysis was performed, interspersed every 10 samples to ensure the reliability and quality of the results.

### 2.5 Preprocessing the Data in MZmine

Data processing in MZmine 3.9.0 was performed using the workflow for direct infusion data on low-resolution equipment.^24^ Mass detection was done within a retention time interval of 0.3–1.3 min and an *m*/*z* interval of 50–1000 Da, with the noise level set at 1.0 × 10^3^ for MS1 and 1.0 × 10^2^ for MS2. The ADAP ChromatogramBuilder algorithm was used for the construction of the chromatogram, employing a minimum of 5 consecutive scans and an intensity limit of 1.0 × 10^3^. The *m*/*z* tolerance for consecutive scans was adjusted to 0.5 Da.

Join Aligner was used with an *m*/*z* tolerance of 0.5 Da and a retention time tolerance of 99999999 min (too large a value to disregard the effect of time on the acquisition of direct infusion spectra), attributing a weight of 0 for retention time and 3 for *m*/*z*. In addition, a row filter was applied to retain variables present in at least three detections. Duplicate traits have been filtered with an *m*/*z* tolerance of 0.25 Da and a retention time tolerance of 99999999 min to eliminate redundancies.

### 2.6 Multivariate Analyses

Both data matrices (ESI­(+) and ESI(−)), from MZmine, were initially normalized by sum, followed by logarithmic transformation in base 10 and autoescalation. Fisher’s discriminant analysis was used as a selection of variables, in order to find the variables responsible for better separating the classes.

For the PLS-DA models, the data matrices in positive and negative modes, composed of 81 samples (44 Fabry and 37 Control), were separated into training (70%) and prediction (30%) sets using the Kennard–Stone algorithm.^25^ The training samples were used to build the classification models, while the prediction samples were later used to evaluate the predictive ability of the models. The optimal number of latent variables was determined by the k-fold cross-validation method, where *k* = 5, based on the Root Mean Square Error of Cross-Validation (RMSECV).^26^

The performance of the classification models was evaluated by the parameter sensitivity, specificity, accuracy, and positive and negative predictive values calculated from the contingency matrix. From the contingency table, the performance parameters of the classification models were evaluated. Sensitivity, estimated from eq 1, is the ability of the model to detect positive samples when they are actually positive.^27–29^

1 $$Sensitivity=\frac{TP}{\left(TP+FN\right)}$$

where TP represents the number of positive samples correctly identified and FN represents the number of positive samples identified as negative, i.e., false-negatives. Specificity (eq 2) describes the ability of the model to identify negative samples.

2 $$Specificity=\frac{TN}{\left(TN+FP\right)}$$

where TN is the number of negative samples correctly identified and FP is the number of negative samples classified as positive, i.e., false-positives. Accuracy (eq 3) is a global parameter for measuring the performance of the model, calculated from the ratio of the number of correctly classified samples to the total number of samples.

3 $$Accuracy=\frac{\left(TP+TN\right)}{\left(TP+TN+FP+FN\right)}$$

Another relevant point in the evaluation of the model was the verification of the predictive positive value (PPV), that is, the probability of a sample being classified as positive when it is actually positive. In order to calculate the probability of a sample classified as negative being really negative, the negative predictive value (NPV) was used. The probabilities of class prevalence are estimated from eqs 4 and 5.

4 $$PPV=\frac{TP}{\left(TP+FP\right)}$$

5 $$NPV=\frac{TN}{\left(TN+FN\right)}$$

The models were built in the MATLAB R2015a environment (MathWorks, USA) using Classification Toolbox 6.0.^30^

## 3 Results and Discussion

Accessible urine samples were divided into two groups: the Case group (of patients diagnosed with FD) (*n* = 45) and the Control group (healthy volunteers) (*n* = 42). The diagnosis of FD was confirmed for the affected patients through genetic testing that identified the presence of mutations in the GLA gene, and all the diagnosed patients had the classic form of the disease since no late onset phenotypes were identified in any of the participants. Regarding gender, there was a predominance of females in both groups, 59.6% in the Case group and 65.4% in the Control group. The mean age of the affected participants was 39.2 ± 16.9 years, while that of the healthy controls was 36.7 ± 10.9 years.^31^

Among the samples that were analyzed, the participants in the Case group were composed of 54.55% female participants and 45.45% male participants. The mean age of the analyzed participants was 40.27 ± 17.26 years, and the percentage of participants being treated with ERT at the time the sample was collected was 61.36%. Of these, 59.26% were being treated with agalsidase-α and 40.74% were being treated with agalsidase-β. However, some of the participants with indications for ERT (namely, numbers 18, 26, 27, 44, and 47 in Table S1) were either not on the medication due to treatment refusal or had been diagnosed too shortly before sample collection.^31^

One aspect that justifies the need for future research in this area is the possibility of relating the obtained data with the use of ERT by the participants. Understanding how the presence of treatment relates to lipid profiles can provide more information regarding the assessment of the response to treatment.

The mass spectra presented below (Figures 1 and 2) were obtained by ESI(±)-LTQ MS in the range 50–1000 Da and represent the relative abundance of ions versus the mass to charge ratio (*m*/*z*). The comparative analysis between the samples from FD patients and the Control group reveals variations in the chemical profile, where different compounds may be present in varying amounts. These variations may indicate the presence of possible biomarkers associated with FD, indicating alterations related to the pathology.

**Figure 1:**
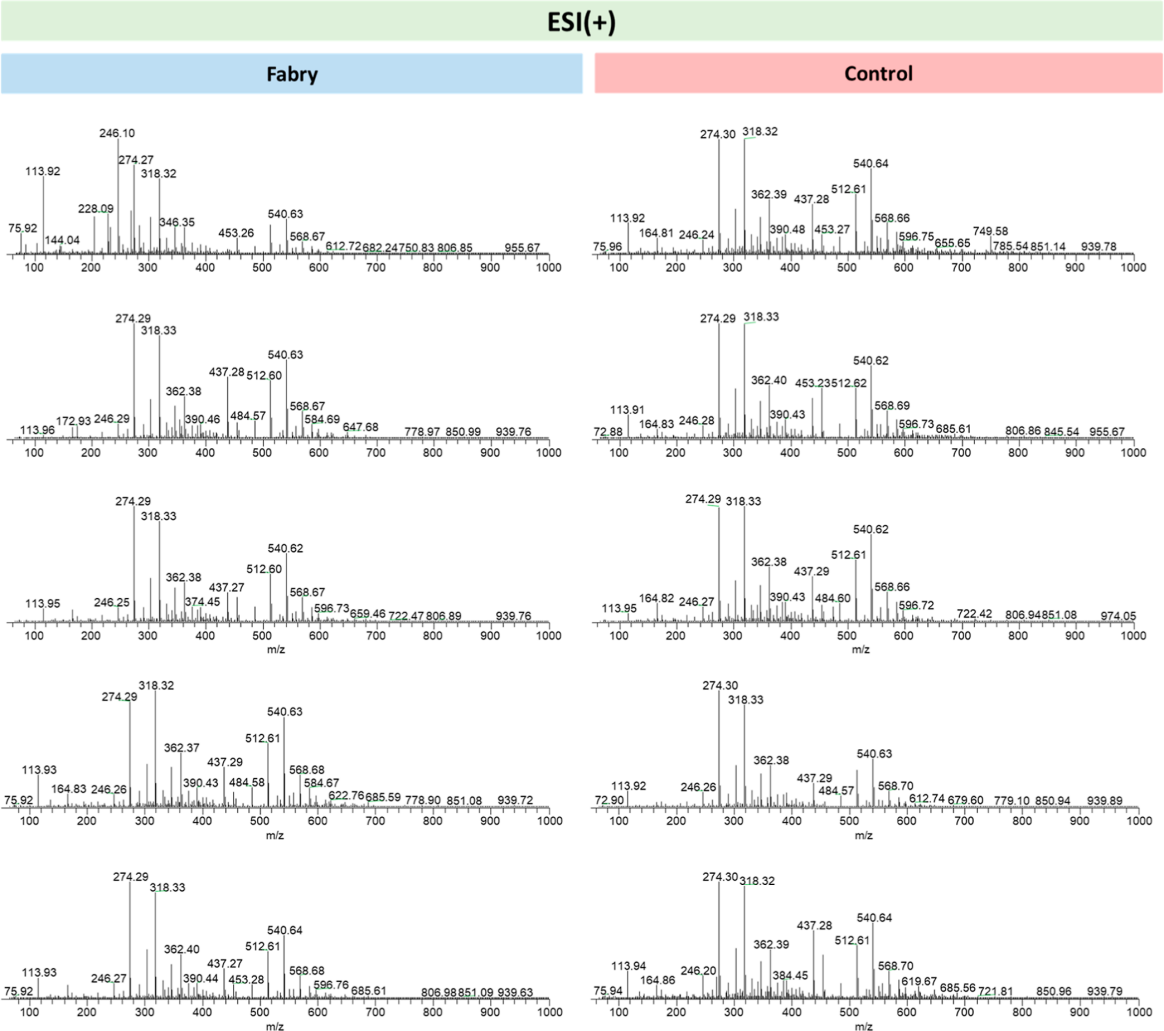
Representative mass spectra obtained by LTQ-MS in the ESI­(+) mode of samples from patients in the Case (left) and Control (right) groups.

**Figure 2:**
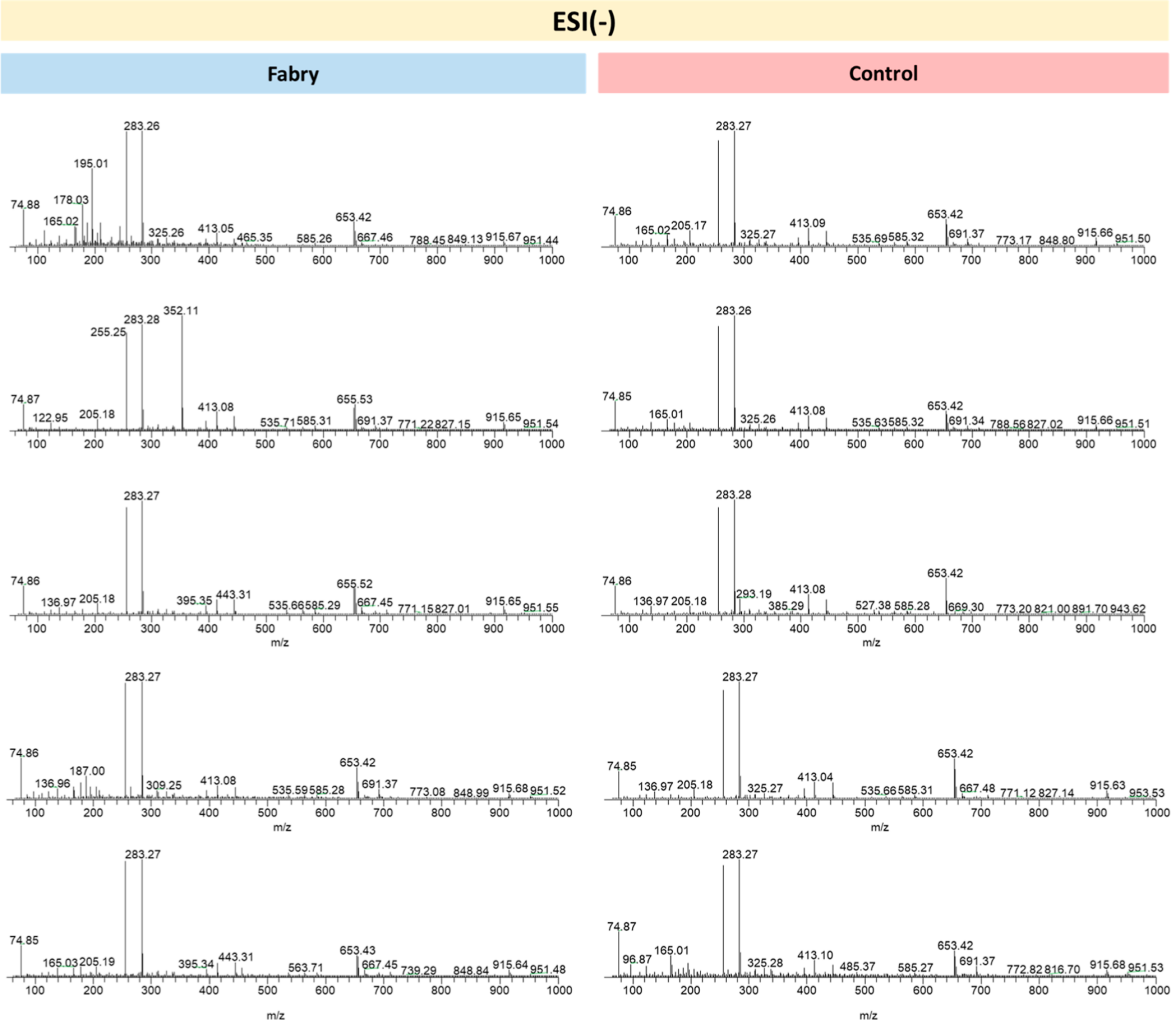
Representative mass spectra obtained by LTQ-MS in the ESI(−) mode from samples from patients in the Case (left) and Control (right) groups.

A major difficulty encountered in the analysis of lipids by MS is related to the extensive isobaric overlap of different molecular lipid species in a sample, in which different compounds may present the same nominal value of *m*/*z*. The most common method used to resolve an isobaric overlap is through the use of chromatographic columns to separate lipids by categories, class, and/or length of the fatty acid chain, which simplifies mass spectra to improve identification and quantification. However, even with chromatographic separation, isobaric lipids still coelute, making qualitative identification problematic.^32^ Given the low resolution of the equipment and bearing in mind that a chromatographic column was not used for separation of molecular species since the goal was not to identify any particular species but to assess the method’s capability to discriminate between affected patients and healthy controls, the precise identification of specific compounds may not have been possible.

Figure 1 represents the mass spectra obtained by LTQ-MS in the ESI­(+) mode of samples from patients in the Case and Control groups. It is possible to observe the similarity between mass spectra of samples that belong to the same group due to the presence of common *m*/*z* values, such as 113.9, 274.2, 318.3, 540.6, and 568.6. It is also possible to observe that samples from affected patients differ from those of healthy controls due to the presence of *m*/*z* values in the samples of the Case group that are not observed in the samples of the Control group, such as 144.0, 228.0, and 346.3. The *m*/*z* value of 172.9 corresponds to a blank solvent (Figure S2a).

Figure 2 represents the mass spectra obtained by LTQ-MS in the ESI(−) mode of the samples from patients in the Case and Control groups. It is possible to observe the similarity between mass spectra of samples that belong to the same group due to the presence of repeating *m*/*z* values, such as 165.0, 653.4, and 655.5. It is also possible to observe that samples from affected patients differ from those from healthy controls due to the presence of *m*/*z* values in the samples of the Case group that are not observed in the samples of the Control group, such as 187.0, 178.03, 195.0, and 352.1. However, unlike the spectra obtained in ESI­(+), most of the peaks corresponded to signals present in the sample blank (Figure S2b), namely, 74.8, 122.9; 136.9, 255.2, 283.2, and 413.0.

Glycosphingolipids are best ionized and most commonly analyzed in the positive mode, especially neutral ones, since these are relatively less ionized in the negative mode relative to their acidic and basic counterparts. In some cases, analysis in the negative mode can be advantageous due to the low chemical background noise and low level of cation adducts formation. In addition, although current approaches to MS provide valuable information on glycosphingolipid structures, they often lack sufficient resolution to distinguish isomeric species with subtle structural differences.^33^ This particularity may have made it impossible to observe a more expressive presence of lipids and other metabolites in the samples analyzed by ESI(−).

Lipids generate class-specific characteristic fragment ions and neutral losses when molecular ions are fragmented under low-energy collision-induced dissociation in MS/MS, with several direct infusion MS methods having been developed for quickly profiling lipid species.^34^ Figures 3 and 4 represent the fragmentation spectra of the highest intensity ions obtained by ESI(±) LTQ-MS.

**Figure 3:**
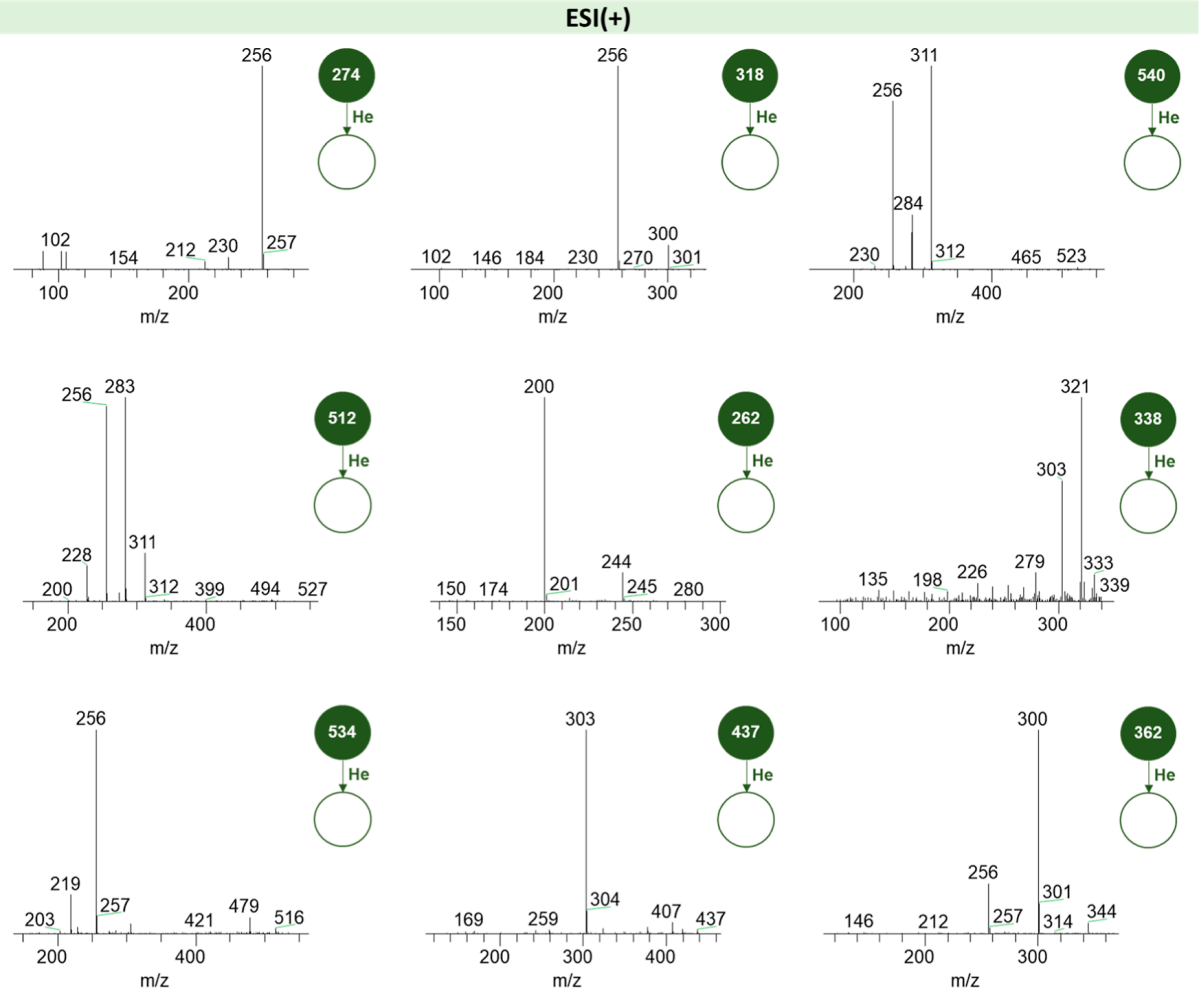
Fragmentation spectra of the highest intensity ions obtained by LTQ-MS in the ESI­(+) mode.

**Figure 4:**
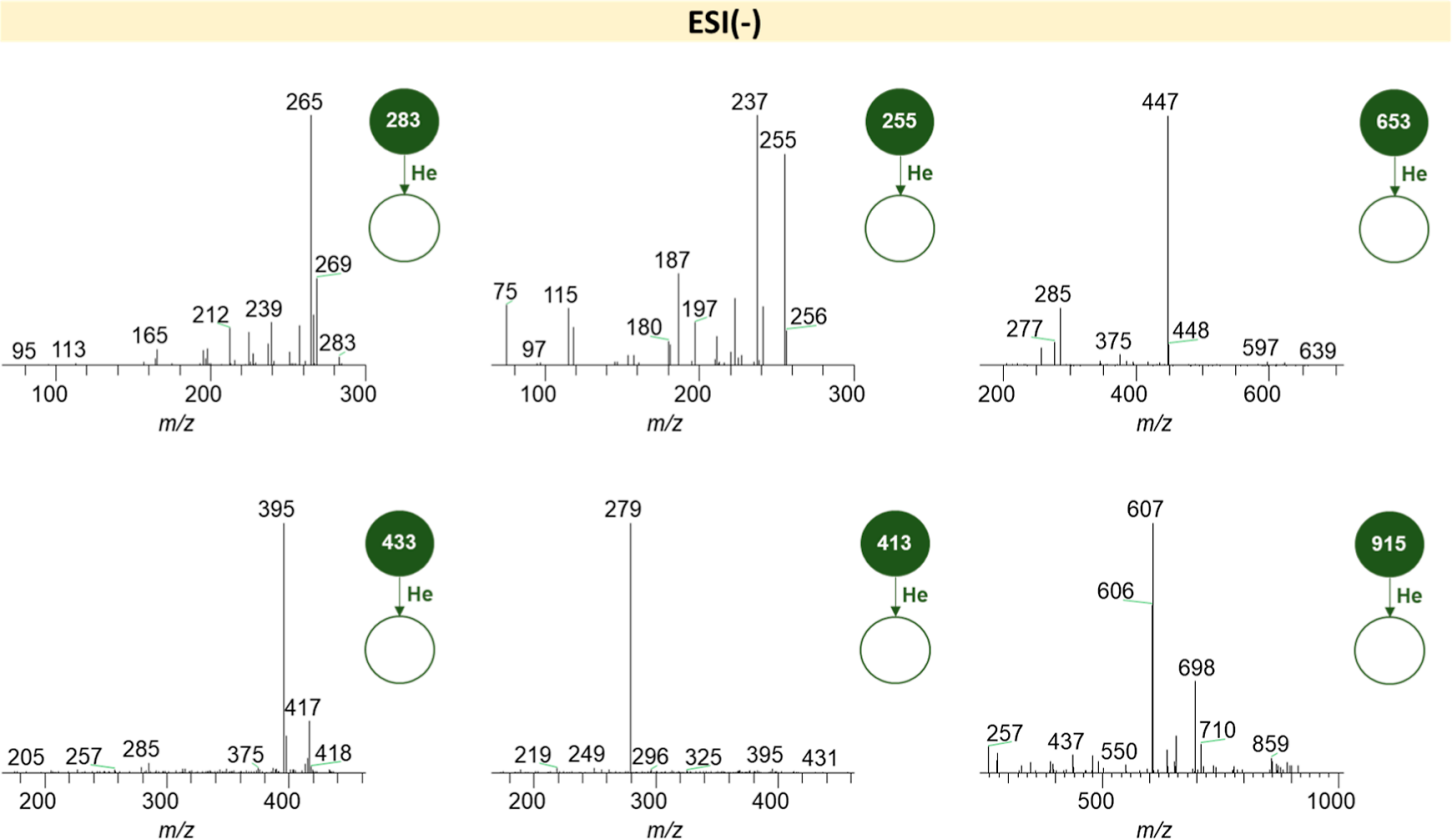
Fragmentation spectra of the highest intensity ions obtained by LTQ-MS in the ESI(−) mode.

By observing Figure 3, it is possible to notice the presence of ions of *m*/*z* equal to 274, 284, 318, 362, and 512 in the positive mode. The latter value may correspond to a molecule of the ceramides class, while the other four values may correspond to sphingoid bases, molecules resulting from the action of the acid ceramidase enzyme over glycosphingolipids.^35,36^ Moreover, the *m*/*z* value equal to 256, visible in more than one fragmentation spectrum, may represent the molecule of protonated palmitamide, the result of the fragmentation of the metabolite ceramide, originated from the catabolism of glycosphingolipids.^37^ Lastly, the *m*/*z* values equal to 512 and 540 may correspond to molecules of lysophospahtidylcholine (14:0 and 16:0, respectively).^38^

By observing Figure 4, it is possible to notice the presence of an ion with *m*/*z* value equal to 698, a possible indicator of glycosphingolipid glucosylceramide, originating from a molecule of lactosylceramide (a Gb3 metabolite) after the elimination of a glucose molecule. Also, the presence of the ion with *m*/*z* equal to 237 may be indicative of the fatty acid chain that composes the structure of lactosylceramide.^39^ Moreover, the *m*/*z* value equal to 180 may pertain to an inositol residue originated from the fragmentation of inositol phosphorylceramide, the *m*/*z* values equal to 256 and 607 may pertain to ceramide molecules (d18:1/18:1, specifically to 607), and the *m*/*z* values equal to 433 and 437 may pertain to lysophosphatidic acid (18:2 and 18:0, respectively).^38,40^

Since lipid concentration tends to vary between individuals, especially in urine samples, reconstituting lipids evaporated by the same volume of organic solvent may result in false negatives since some lipids at relatively low concentrations may not be detected or quantified because their concentrations are below the detection or quantification limits of the equipment.^38^ This limitation may have impaired the visualization of the lipid classes of interest in the samples reconstituted for analysis. In addition, it is possible that the use of ERT by a large part of the participants in the Case group affected the lipid concentration of the samples collected, therefore making the enzymatic substrates often used as biomarkers of FD absent and undetectable.

Although it was possible to observe the presence of glycosphingolipid metabolites and precursor molecules in the extracted samples, the main urinary biomarker lyso-Gb3 and its analogues were not identified. Studies published in the area of FD diagnostic research aimed at identifying and quantifying lyso-Gb3 and its related analogues in urine revealed the following *m*/*z* values for the molecules and their main fragments under analysis by means of ESI­(+) MS/MS: 786.3 → 282.3; 758.3 → 254.3; 774.3 → 252.3; 784.3 → 280.3; 800.3 → 278.3; 802.3 → 280.3; 820.3 → 334.3; and 836.3 → 339.3.^41,42^

The mechanism of generation of Lyso-Gb3 by nonspecific action of acid ceramidase has been proposed but is not yet fully understood.^43^ Glycosphingolipids are composed of multiple isoforms based on modifications of fatty acid chains, and little is understood about the biological relevance of each one. It is known, however, that lyso-Gb3 analogues are more prevalent in urine than lyso-Gb3 itself. Therefore, in order to better discriminate patients affected by FD, it may be advantageous to increase the range of *m*/*z* to include molecules with a mass greater than 1000 Da, such as galabiosilceramide (Ga2) and its isoforms.^44^

In order to evaluate whether an extraction method with organic solvents followed by ESI(±)-LTQ MS analysis could distinguish between healthy controls and patients affected by FD, multivariate analysis was performed on the data for the spectra obtained in both positive and negative modes through PCA and PLS-DA.

For the PCA performed from the spectra obtained in positive (ESI­(+)), negative (ESI(−)), and combined (ESI(±)) modes, Figure 5 displays the scatter plots between the selected components. In the PCA of the samples read in the positive mode, PC1 and PC2 explain, respectively, 24.15% and 7.33% of the variation in the data set. In the PCA of the samples read in the negative mode, PC1 and PC2 explain, respectively, 31.71% and 8.73% of the variation in the data set. In the PCA of the samples in the combined mode, PC1 and PC2 explain, respectively, 13.44% and 10.06% of the variation in the data set.

**Figure 5:**
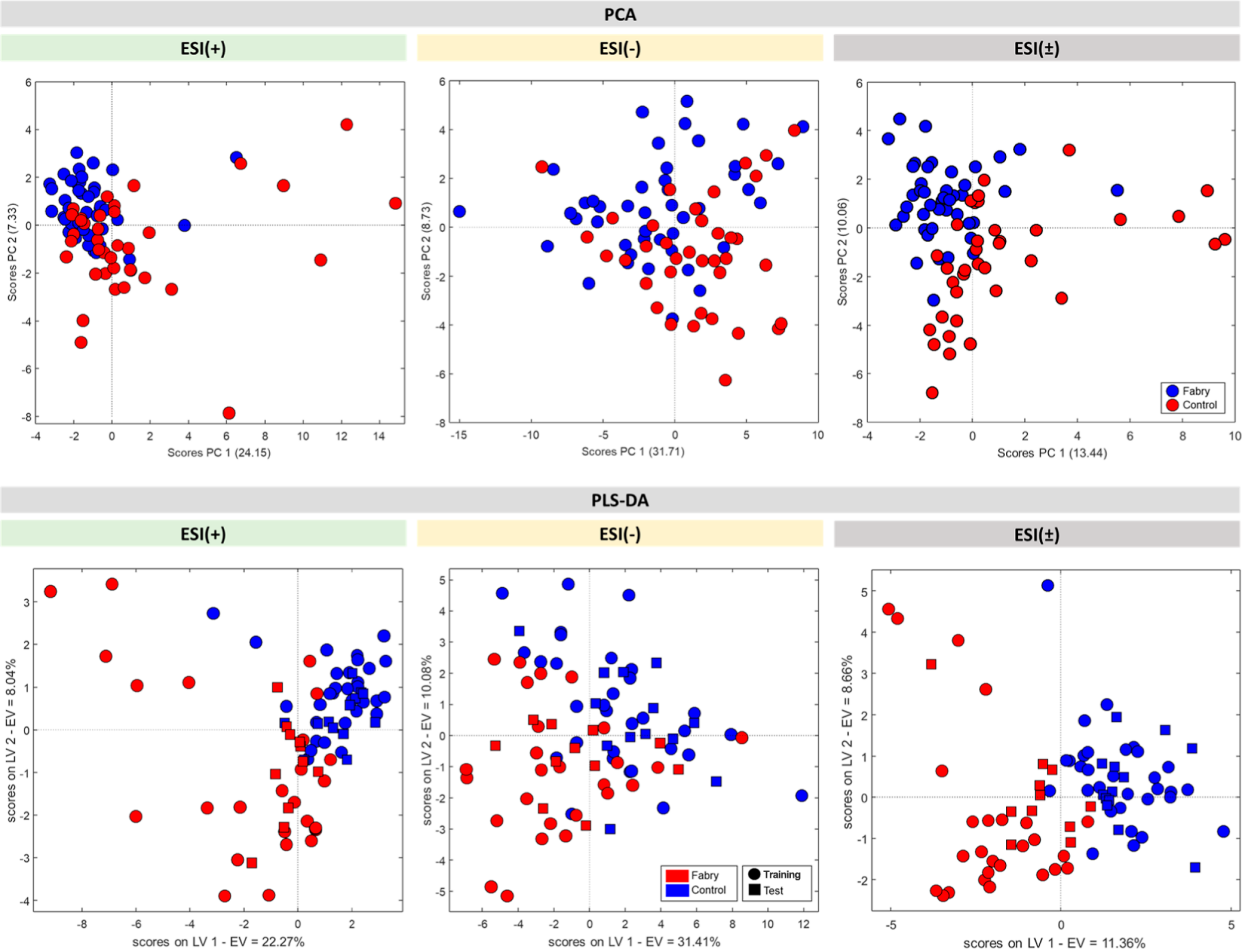
PCA and PLS-DA scatter plots of samples obtained from ESI(±)-LTQ MS. The PC1 and PC2 components and the latent variables LV1 and LV2 represent the percentages of the variation in the data set. The red dots represent samples from the Case group and the blue dots represent samples from the Control group. The circles represent training samples (used in the construction of the classification models) and the squares represent test samples (used to evaluate the quality of the model).

Although the PCAs performed were not able to completely separate the two groups, a trend of separation between Fabry and Control samples can be observed due to the tendency of Fabry samples to be located in the positive portion of PC2 and Control samples to be located in the negative portion of PC2. In addition, in the positive mode, samples from both groups were more concentrated in the negative portion of PC1, while in the negative mode, there was no concentration throughout PC1. In the combined mode (ESI(±)), Fabry samples tended to be positioned in the negative region of PC1 and the positive region of PC2, while Control samples were located in the positive region of PC1 and the negative region of PC2. The combination of both modes resulted in better separation of the groups. This highlights the advantage of integrating both positive and negative ionization modes as an alternative to enhance the distinction between the samples.

For the PLS-DA performed from the spectra obtained in positive and negative modes, Figure 5 also displays the scatter plots between the selected latent variables. In the PLS-DA of the samples read in the positive mode, the latent variables LV1 and LV2 explain, respectively, 22.27% and 8.04% of the variation in the data set. In the PLS-DA of the samples read in the negative mode, the latent variables LV1 and LV2 explain, respectively, 31.41% and 10.08% of the variation in the data set. In the PLS-DA of the combined mode, the latent variables LV1 and LV2 explain, respectively, 11.36% and 8.66% of the variation in the data set.

According to the data obtained from PLS-DA, it was possible to maximize the class separation so that a significant differentiation of the groups could be observed. Mostly, in the positive mode, the Control samples were more concentrated on the positive portion of variables 1 and 2, unlike the Fabry samples that seem to have been more concentrated on the negative portions of LV1 and LV2. Generally speaking, it can be observed that PLS-DA provided a better separation of the two groups along the axes of the latent variables.

The graphs in Figure 6 represent the distribution analysis carried out for the PLS-DA. In the graphs on the left, the samples were related according to the probability of each one belonging to one group or the other, with the probability ranging from 0 to 1. These results demonstrate the separation of the Case and Control groups in both the training and test sets. Ideally, all samples of the same color would be on the same side and as close as possible to the upper (100% probability of belonging to the Case group) and lower (100% probability of belonging to the Control group) borders. However, it was possible to observe that some samples (both training and test) were incorrectly classified along the distribution in both positive and negative modes.

**Figure 6:**
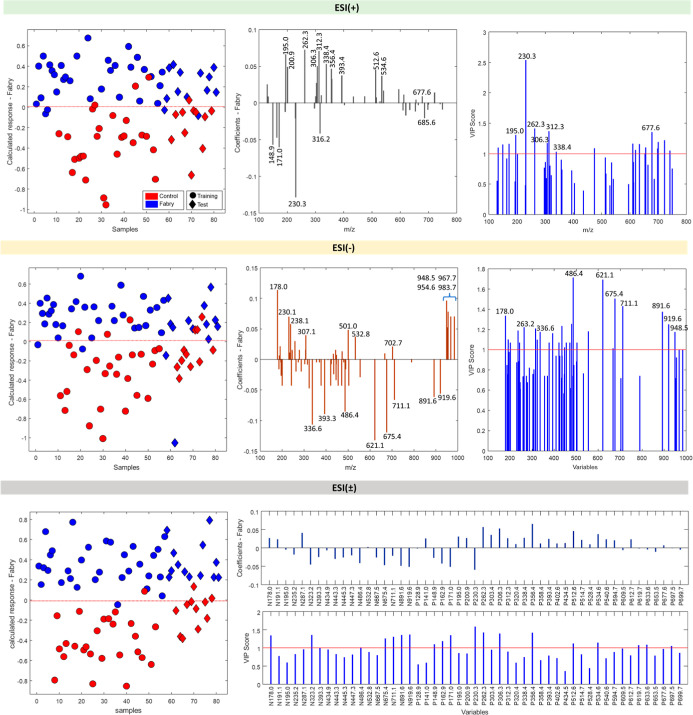
Calculated response plot, coefficients, and VIP scores of PLS-DA of lipid profiles, differentiating the Control and Case (Fabry) groups. The red dots represent samples from the Case group, and the blue dots represent samples from the Control group. The circles represent training samples (used in the construction of classification models) and the rhombuses represent test samples (used to evaluate the quality of the model). The dashed red line represents the limit set by PLS-DA.

The graphs on the right indicate the *m*/*z* values that contributed the most to the separation of the groups, with the most important for the Case group being those with greater intensity on the positive side and the most important for the Control group being those with greater intensity on the negative side. For the samples read in the positive mode (6a), the multivariate analysis indicated that the most important *m*/*z* values for the Case group are as follows: 195.0, 200.9, 262.3, 312.3, 338.4, 356.4, 393.4, 512.6, and 534.6. For the samples read in the negative mode (6b), the multivariate analysis indicated that the *m*/*z* values that most contributed to the separation of the groups are as follows: 178.0, 230.1, 238.1, 307.1, 501.0, 532.8, 702.7, 948.5, 954.6, 967.7, and 983.7. It would seem that no major *m*/*z* value appears simultaneously in the positive and negative modes.

Interestingly, the most important *m*/*z* values, as well as those obtained for the fragmentation spectra, were not compatible with those of the lyso-Gb3 biomarker and its isoforms listed in the literature. It is possible that the low number of samples has limited the ability to demonstrate the full sensitivity of the MS technique as a diagnostic method and that the application of a low-resolution spectrometer has failed to provide a more comprehensive lipidomic analysis.^35^ However, further research is required in order to determine if these values may represent potential biomarkers of FD that have yet not been described or validated.

Analyzing the results of the classification metrics (Table 1), the classification model obtained 85% sensitivity and 91% specificity for the Case class of the test set of samples read in the positive mode, whereas, in the analysis in the negative mode, the classification model obtained 85% sensitivity and 73% specificity for the Case class of the test set. The PPV and NPV values of the test set were higher for the samples read in the positive mode (92% and 93%, respectively) than for the samples obtained in the negative mode (79% and 80%, respectively). Accuracy was also higher in the test set of the positive mode (88%) than in the test set of the negative mode (79%). Therefore, according to these results, it is possible to affirm that the analysis in the positive mode showed a better ability to discern the samples in relation to the negative mode, correctly marking samples as belonging to their classes of origin more frequently and with a lower error rate (12% in the positive and 21% in the negative for the test set).

**Table 1:** Classification Model Metrics Results^a^

Model	FD	LV	Set	Class	Sensitivity	Specificity	Error rate	PPV	NPV	Accuracy
ESI(+)	30%	2	Training	Case	94%	88%	9%	91%	92%	91%
				Control	88%	94%				
			Test	Case	85%	91%	12%	92%	83%	88%
				Control	91%	85%				
ESI(−)	35%	4	Training	Case	94%	88%	9%	91%	92%	91%
				Control	88%	94%				
			Test	Case	85%	73%	21%	79%	80%	79%
				Control	73%	85%				
ESI(±)	35%	2	Training	Case	97%	96%	4%	97%	96%	96%
				Control	96%	97%				
			Test	Case	100%	82%	9%	87%	100%	92%
				Control	82%	100%				

a FD, Fisher’s discriminant. LV, latent variables. PPV, predictive positive value. NPV, negative predictive value. Acc, accuracy.

However, in comparison with the individual modes, the combined mode (ESI(±)) achieved higher classification metrics. For the Case test set, the combined mode resulted in 100% sensitivity, 82% specificity, 87% PPV, 100% NPV, 9% error rate, and 92% accuracy. These results confirm that the combined mode provides better overall classification accuracy with higher sensitivity, specificity, and PPV/NPV, compared to both individual modes. The combined mode enhanced the ability to correctly distinguish between the groups, thereby optimizing the classification performance.

Regarding the fragmentation spectra of the main discriminant compounds identified in the analysis of VIP score from the PLS-DA model modes (Figure 7), the observed ions (e.g., *m*/*z* 262, 306, 312, and 356) in the positive mode showed consecutive losses of 18 Da (H_2_O) and 44 Da (CO_2_), indicating the presence of hydroxyl and carboxyl functional groups.^40^ These fragmentation patterns are compatible with fragments of lipids such as glycosphingolipids or oxidized fatty acids, both potentially present in the urine of patients with Fabry disease. The characteristic lysosomal accumulation of glycosphingolipids in this pathology may result in the formation and subsequent urinary excretion of various lipid metabolites derived from degradation or oxidation processes.

**Figure 7:**
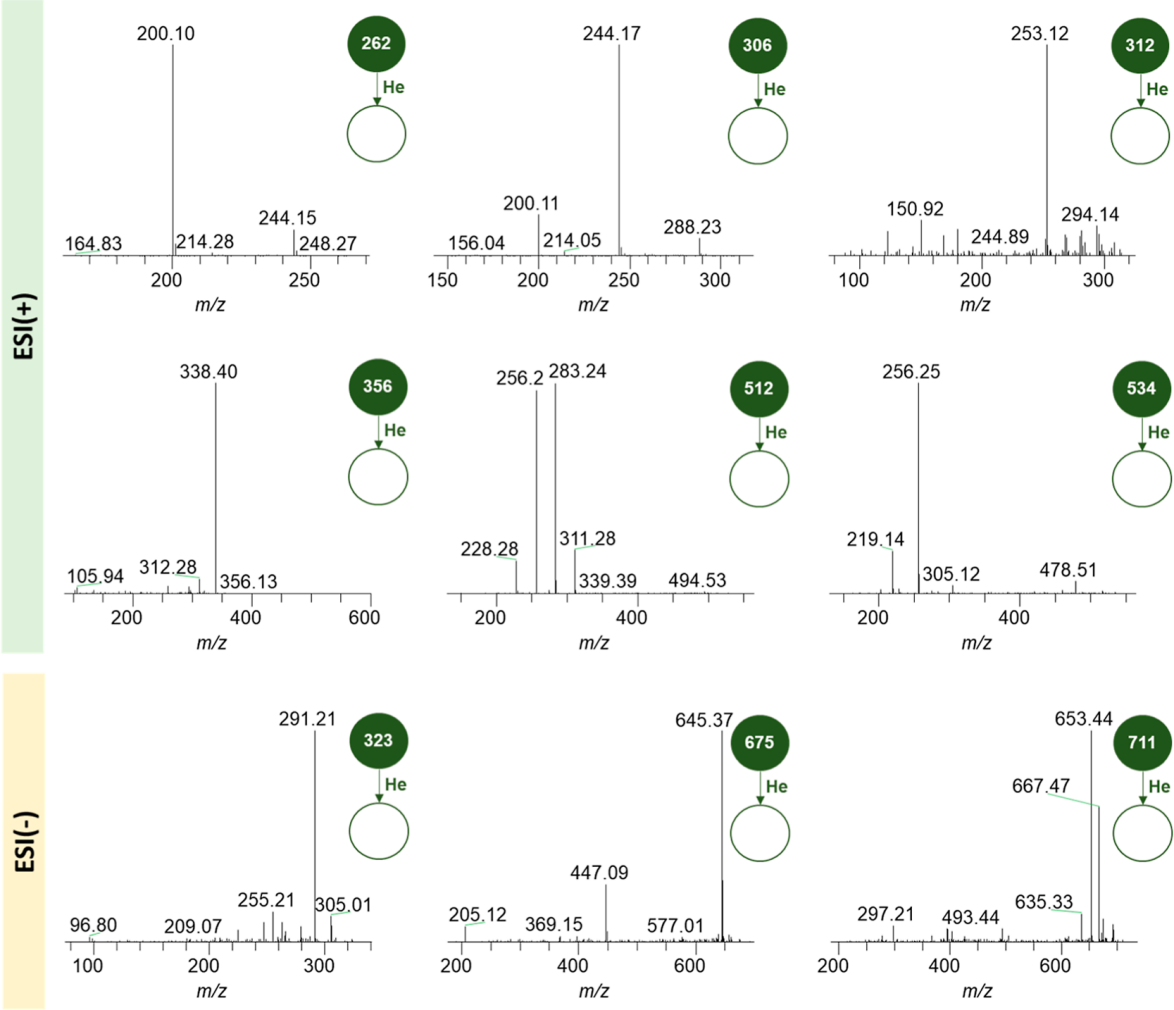
Fragmentation spectra of the main compounds identified as discriminant by the PLS-DA model from ESI­(+) and ESI(−) ionization modes.

The choice of urine as a specimen for lipid analysis proved to be able to differentiate the Case and Control groups, being a more viable alternative than other biological fluids for a number of reasons. Urine can be collected noninvasively and can be easily stored and handled, and biomarkers of interest for FD are present in samples collected from affected individuals. That being said, the reduced abundance of most biomarkers in biologically accessible samples obtained from patients with lysosomal storage diseases (LSDs) is usually a hindrance for the majority of screening techniques since nontargeted approaches sacrifice sensitivity and precise quantification for the possibility of building a metabolic profile or fingerprint. However, once a certain biomarker has been identified and validated, its directed measurement can compensate for abundance issues.^45^

Targeted approaches have been applied to potentially diagnose FD using MS methodologies and have been successful using relatively large sample sets.^46^ One recent study in particular established that lyso-Gb3, analyzed by LC–MS/MS, was more successful in diagnosing women affected by FD.^47^ However, this biomarker cannot be applied in younger patients since age has a significant impact on lyso-Gb3 concentration.^48^ More recently, it has been demonstrated through LC–MS/MS that there are potential serum and urine markers in the female FD cohort.^44^ This study was the first to apply orthogonal PCA and PLS-DA strategies, in addition to standard univariate comparisons, in order to identify potential biomarkers of FD.^46^

According to Heywood et al.,^44^ urinary lyso-Gb3 appears to perform worse than serum lyso-Gb3, likely due to the low concentration of lyso-Gb3 in urine. Also, according to the authors, serum lyso-Gb3 seems to be more specific for women with symptomatic FD and acts as a marker of disease severity but has limited utility in the detection of asymptomatic heterozygous women. Finally, the authors conclude by proposing that it may be more advantageous to combine lyso-Gb3 analysis with other glycosphingolipids, particularly for women with an uncertain diagnosis and that this could be easily incorporated into LC–MS analysis in chemical pathology laboratories.

Traditional methods for analyzing amphiphilic glycolipids are often time-consuming and require deacylation and derivatization steps. However, faster and more specific methods utilizing protein precipitation and liquid or solid phase extraction in combination with MS and flow injection analysis have already been described. It has been observed that it may be advantageous to omit the chromatography step and instead focus on the extraction and purification steps during sample preparation in routine robust assays.^49^

In the future, during the analysis of data obtained in higher-resolution equipment, adding the orthogonal PLS-DA multivariate analysis, such as Heywood et al.^44^ may lead to a more clarifying discrimination of the two groups. However, according to a review by Percival et al.,^46^ the use of metabolomic approaches in the clinical diagnosis and monitoring of lysosomal storage disorders is still a novelty with a wide range of future opportunities. Required future developments include systematic sample collection and handling; further validation of potential biomarkers selected based on monitoring of their levels in biofluids and the assessment of clinical responses to treatment; detailed correlations of biomarker concentrations with disease severity, age, gender, and diet, among other variables; and a more detailed review to ensure that the biomarkers identified are specific to a particular lysosomal storage disorder.

## 4 Conclusion

The extraction method and analytical technique used allowed the detection of metabolites from the class of interest. While the PCA did not show a clear distinction between the Case and Control groups, a slight trend toward separation was observed, especially in the combined mode (ESI(±)) plot. In contrast, the PLS-DA results show a better separation between the groups, indicating the relevance of using spectrometric techniques associated with multivariate analysis methods for diagnosing the disease. The analyses performed in the positive ionization mode exhibited superior sensitivity, specificity, accuracy, and a lower error rate than those performed in the negative ionization mode. In comparison to the individual modes, the combined mode showed a significant improvement, achieving higher classification metrics for the test set (92% accuracy for ESI(±), 88% for ESI­(+), and 79% for ESI(−)). The combined mode resulted in better overall separation between the groups, further emphasizing the advantage of integrating both ionization modes to enhance the model’s ability to accurately distinguish between the groups. This study demonstrates the effectiveness of direct infusion in a low-resolution mass spectrometer, combined with chemometric techniques, in efficiently discriminating samples from two patient groups for the diagnosis of Fabry disease. However, further analyses using high-resolution mass spectrometry and LC separation are necessary to enable identification of the main discriminants.

## Supplementary Material



## Notes

Table of clinical data from patients diagnosed with Fabry Disease, including gender, age, enzyme replacement therapy (ERT) usage, recombinant enzyme administered, and signs and symptoms, and mass spectra of blank samples acquired by ESI­(+) and ESI(−) modes (PDF)
