# Supplementary material for: Untargeted Lipidomics in Fabry Disease of Urine Samples by Low-Resolution Flow Injection Mass Spectrometry (ESI(±)-LTQ MS)
Source: ACS Omega. 2025 Jun 27;10(26):27869–82. doi: 10.1021/acsomega.5c00894 (PMC12242645; doi:10.1021/acsomega.5c00894)
Supplement: Supplementary file 1 [file ao5c00894_si_001.pdf]

Untargeted lipidomics in Fabry disease of urine samples by low resolution flow injection mass spectrometry (ESI(±)-LTQ MS)

Rafael Arruda Foletto[a,b,c]; Augusto Santos Borges[a,b]; Larissa Campos Motta[c]; Marcos Valério Vieira Lyrio[c]; Carolina Teles Baretto[d]; Luciene Cristina Gastalho Campos[d]; Paulo Roberto Filgueiras[c]; Valério Garrone Barauna[a]; Wanderson Romão[b,c,*].

[a]Laboratory of Molecular Physiology and Artificial Intelligence, Department of Physiological Science, Federal University of Espírito Santo (UFES), Vitória, ES, Brazil.

[b]Federal Institute of Espírito Santo (IFES), Vila Velha, ES, Brazil.

[c]Laboratory of Petroleomics and Forensic Chemistry, Chemistry Department, Federal University of Espírito Santo (UFES), Vitória, ES, Brazil.

[d]Postgraduate Program in Health Sciences, State University of Santa Cruz, Ilhéus, BA, Brazil.

Author for correspondence: Wanderson Romão

Federal Institute of Espírito Santo (IFES)
Av. Min. Salgado Filho, 1000 – Vila Velha, ES

wandersonromao@gmail.com

S1: table of information from patients diagnosed with FD. Gender (M, male; F, female); age in years; whether or not made use of ERT (Y, yes; N, no); which enzyme was administered in ERT; signs and symptoms. [a]Dialysis; [b]Kidney transplantation; CKD, chronic kidney disease; ESKD, end-stage kidney disease; GI, gastrointestinal; LVH, left ventricular hypertrophy; N/A, not available.

Patient Number	Gender	Age (Years)	ERT	Recombinant Enzyme	SignsandSymptoms
1	F	55	Y	Agalsidase β	Acroparesthesis
2	M	29	Y	Agalsidase β	Acroparesthesis<br>Angiokeratoma<br>Hypohidrosis<br>Proteinuria
4	M	15	Y	Agalsidase β	Acroparesthesis<br>Angiokeratoma
6	F	64	N	N/A	N/A
9	F	28	N	N/A	N/A
10	M	16	Y	Agalsidase β	Acroparesthesis<br>Angiokeratoma<br>Biopsy<br>Hypohidrosis
11	M	18	Y	Agalsidase β	Acroparesthesis<br>Angiokeratoma<br>Biopsy<br>Hypohidrosis
12	F	40	N	N/A	N/A
13	F	36	N	N/A	N/A
14	F	39	Y	Agalsidase β	Acroparesthesis
15	F	31	N	N/A	N/A
16	F	18	N	N/A	N/A
17	F	71	N	N/A	Acroparesthesis
18	F	40	N	N/A	Acroparesthesis<br>Biopsy
19	F	51	Y	Agalsidase α	Acroparesthesis<br>Hypohidrosis
24	M	59	Y	Agalsidase α	Acroparesthesis<br>Hypohidrosis<br>Microalbuminuria
25	M	66	Y	Agalsidase α	Acroparesthesis<br>Hypohidrosis<br>Microalbuminuria<br>LVH
26	M	11	N	N/A	Acroparesthesis<br>Hypohidrosis<br>Microalbuminuria
27	M	12	N	N/A	Acroparesthesis<br>Hypohidrosis<br>Microalbuminuria
28	F	61	Y	Agalsidase α	Acroparesthesis<br>Hypohidrosis<br>Proteinuria
29	F	49	Y	Agalsidase α	Acroparesthesis<br>Hypohidrosis
30	F	55	Y	Agalsidase α	Acroparesthesis<br>Hypohidrosis<br>Microalbuminuria

31	M	55	Y	Agalsidase α	Acroparesthesis<br>Hypohidrosis
32	F	41	Y	Agalsidase α	Acroparesthesis<br>Hypohidrosis<br>Microalbuminuria
33	M	55	Y	Agalsidase α	Acroparesthesis<br>Hypohidrosis
34	F	47	N	N/A	N/A
35	M	28	Y	Agalsidase β	Acroparesthesis<br>Angiokeratoma<br>ESKD[a]
36	M	20	Y	Agalsidase β	Acroparesthesis<br>Angiokeratoma
37	M	42	Y	Agalsidase β	Acroparesthesis<br>Angiokeratoma<br>ESKD[b]
38	M	43	Y	Agalsidase β	Acroparesthesis
39	F	68	N	N/A	N/A
40	F	64	Y	Agalsidase β	Acroparesthesis<br>Angiokeratoma<br>CKD
41	F	38	Y	Agalsidase α	ESKD[a]
42	F	37	N	N/A	N/A
43	F	15	N	N/A	N/A
44	M	24	N	N/A	N/A
45	F	46	Y	Agalsidase β	Acroparesthesis<br>Biopsy
46	M	50	Y	Agalsidase β	Acroparesthesis<br>Angiokeratoma<br>Hypohidrosis<br>ESKD[b]
47	M	20	Y	Agalsidase β	Acroparesthesis<br>Angiokeratoma<br>Hypohidrosis
48	F	64	N	N/A	Proteinuria
49	F	25	Y	Agalsidase β	Acroparesthesis<br>Angiokeratoma<br>Cornea Verticillata<br>Hypohidrosis<br>White Matter Lesion
50	M	34	Y	Agalsidase β	Acroparesthesis<br>Angiokeratoma<br>Hypohidrosis<br>ESKD[b]<br>LVH
51	M	35	N	N/A	Acroparesthesis<br>Angiokeratoma<br>Hypohidrosis<br>GI symptoms<br>CKD
52	M	57	Y	Agalsidase α	GI symptoms<br>CKD

S2 – Mass spectra from blank of ESI(±) samples.

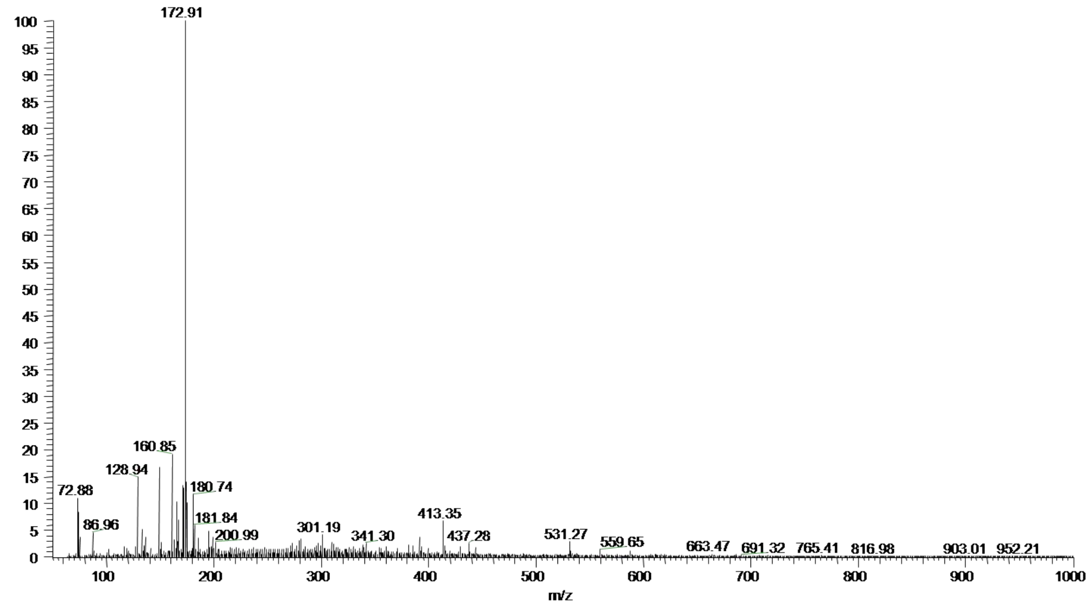

S2a: Mass spectra from blank of ESI(+) samples.

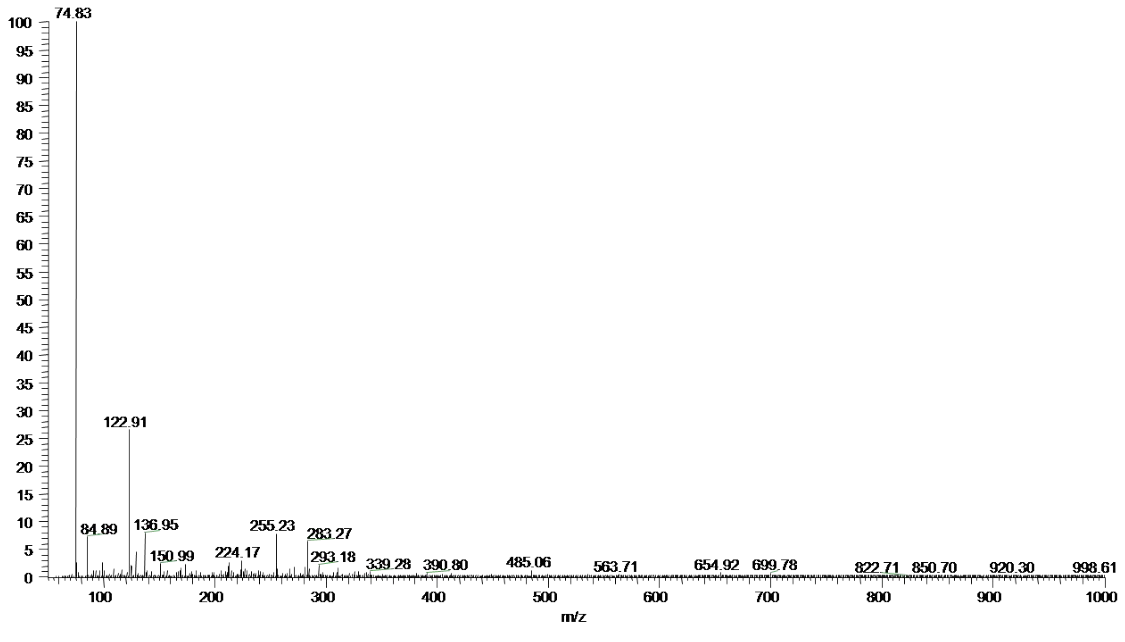

S2b: Mass spectra from blank of ESI(-) samples.
